# Efficient Selection of Gaussian Kernel SVM Parameters for Imbalanced Data

**DOI:** 10.3390/genes14030583

**Published:** 2023-02-25

**Authors:** Chen-An Tsai, Yu-Jing Chang

**Affiliations:** Division of Biometry, Department of Agronomy, National Taiwan University, Taipei 106216, Taiwan

**Keywords:** support vector machine, imbalanced datasets, threshold adjustment, parameter selection

## Abstract

For medical data mining, the development of a class prediction model has been widely used to deal with various kinds of data classification problems. Classification models especially for high-dimensional gene expression datasets have attracted many researchers in order to identify marker genes for distinguishing any type of cancer cells from their corresponding normal cells. However, skewed class distributions often occur in the medical datasets in which at least one of the classes has a relatively small number of observations. A classifier induced by such an imbalanced dataset typically has a high accuracy for the majority class and poor prediction for the minority class. In this study, we focus on an SVM classifier with a Gaussian radial basis kernel for a binary classification problem. In order to take advantage of an SVM and to achieve the best generalization ability for improving the classification performance, we will address two important problems: the class imbalance and parameter selection during SVM parameter optimization. First of all, we proposed a novel adjustment method called b-SVM, for adjusting the cutoff threshold of the SVM. Second, we proposed a fast and simple approach, called the Min-max gamma selection, to optimize the model parameters of SVMs without carrying out an extensive k-fold cross validation. An extensive comparison with a standard SVM and well-known existing methods are carried out to evaluate the performance of our proposed algorithms using simulated and real datasets. The experimental results show that our proposed algorithms outperform the over-sampling techniques and existing SVM-based solutions. This study also shows that the proposed Min-max gamma selection is at least 10 times faster than the cross-validation selection based on the average running time on six real datasets.

## 1. Introduction

In recent years, medical data mining has gained recognition, and especially, the development of the class prediction model has been of great interest. The classification of medical datasets arises in many applications, such as medical diagnostic tests of diseases and gene expression tests. Medical diagnosis is used to find out the diseases of patients based on the given symptoms and physical examinations. Gene expression tests are to predict the probability of diseases based on the genes associated with the phenotype or disease. About diseases such as liver cancer, lung cancer, breast cancer, and gastric cancer, the early diagnosis or prediction of these diseases are pretty vital, because they can prevent or stop an outbreak and even save precious time. Hence, developing a powerful prediction model is considered as a primary task for medical data mining. However, medical datasets often have the imbalanced classes distribution problem, which means positive outcomes are rare compared to the negative outcomes, and what we are interested in is the minority class rather than the majority class.

Imbalanced datasets are considered as critical issues in data mining and machine learning. The conventional classifiers generally have a high prediction for the majority class but fail to detect the minority class, because they are designed for maximizing the overall accuracy and assume that the costs misclassification are equal.

The support vector machine (SVM) is the most popular classifier algorithm and has been proven to outperform other classification methods when dealing with high-dimensional datasets and numerical features [1]. Because it can deal with nonlinear and high-dimensional problems, it has a good performance for many different datasets. The standard SVM is formulated as follows:

Primal problem:

$$\begin{array}{cc} \min_{w} & \frac{1}{2}{∥w∥}^{2}+C\sum_{i=1}^{N}\xi_{i}\\ \mathrm{subject}\;\mathrm{to} & y_{i}\left(w^{T}\phi \left(x_{i}\right)+b\right)\geq 1-\xi_{i};\\ \; & \xi_{i}\geq 0,i=1,2,\ldots ,N. \end{array}$$

$$y_{i}=\left\{\begin{array}{cc} +1 & \;\mathrm{if}\;x_{i}\in class\left(+1\right);\\ -1 & \;\mathrm{if}\;x_{i}\in class\left(-1\right), \end{array}\right.$$

where w is an orthogonal vector to the hyperplane $w^{T}\phi \left(x_{i}\right)+b=0$, *C* is the cost of misclassification, b∈R is the bias, $\phi (x_{i})$ is a mapping function, and $\xi_{i}$ is a slack variable. Slack variables measure the error that includes the data points on the wrong side of the hyperplane or within the margin. Hence, the classifier can be written as $\hat{f}\left(x_{i}\right)=w^{T}\phi \left(x_{i}\right)+b$, and the predicted label is $sgn[\hat{f}\left(x_{i}\right)]$.

Because it is difficult to solve the primal problem directly when the mapping function is not the identity function, we need to convert the primal problem into the dual problem:

Dual problem:

$$\begin{array}{cc} \max_{\alpha } & \sum_{i=1}^{N}\alpha_{i}-\sum_{i,j=1}^{N}\alpha_{i}\alpha_{j}y_{i}y_{j}K\left(x_{i},x_{j}\right)\\ \mathrm{subject}\;\mathrm{to} & 0\leq \alpha_{i}\leq C;\\ \; & \sum_{i=1}^{N}y_{i}\alpha_{i}=0,i=1,2,\ldots ,N.\\ \; & K\left(x_{i},x_{j}\right)=\phi {\left(x_{i}\right)}^{T}\phi \left(x_{j}\right), \end{array}$$

where $\alpha_{i}$ is a Lagrange multiplier, and $K(x_{i},x_{j})$ is a kernel function. Common choices for kernel functions are linear function $K\left(x_{i},x_{j}\right)={x_{i}}^{T}x_{j}$, polynomial function $K\left(x_{i},x_{j}\right)={\left({x_{i}}^{T}x_{j}+c\right)}^{d}$, and Gaussian radial basis function $K\left(x_{i},x_{j}\right)=\exp^{-\gamma {∥x_{i}-x_{j}∥}^{2}}$. In particular, Gaussian radial basis function is very popular in the SVM. In general, $K(x_{i},x_{j})$ can be considered as the similarity measurement between two data points $x_{i}$ and $x_{j}$. In the dual standard SVM, the classifier can be rewritten as $\hat{f}\left(x_{i}\right)=\sum_{j=1}^{N}\alpha_{j}y_{j}K\left(x_{i},x_{j}\right)+b$.

Although the standard SVM is a powerful tool for classification, it still has some drawbacks:

1. The hyperplane used in the SVM algorithm will skew toward the minority class if the training dataset is imbalanced. The objective of the conventional SVM is to maximize the overall accuracy and an equal misclassification cost is assumed in the classifiers.
2. The performance of the SVM highly depends on the parameter selection and its kernel selection. In general, it can be very time consuming to optimize its parameters by using a grid search.

The SVM is based on the structural risk minimization (SRM) and aims to maximize the margin and minimize the misclassification error. As a consequence, in order to lower $\sum_{i=1}^{N}\xi_{i}$ (misclassification error), the hyperplane will skew toward the minority class in the imbalanced dataset so that the SVM easily misclassifies the new observations to the majority class. So far, the common solutions to this problem are re-sampling, cost-sensitive learning, and a threshold adjustment.

Re-sampling is used to modify the dataset to improve its balance, and it can be categorized into two groups: under-sampling (such as a one-sided selection [2]) and over-sampling (such as SMOTE [3] and borderline SMOTE [4]). Although re-sampling is the easiest way to improve the performance, it still has some drawbacks. Under-sampling may lose some valuable information, and over-sampling will increase the completion time.

Cost-sensitive learning [5] is to adjust the misclassification cost between the majority class and minority class, and the ratio of two costs can be determined by the inverse of the imbalanced ratio (IR), which is defined as the proportion samples in the number of the majority class to the minority class [6], PSO algorithm [7], and information entropy [8]. However, some researchers do not recommend cost-sensitive learning. They believe that the improving effect will be limited because the Karush–Kuhn–Tucker (KKT) conditions take the penalty constants as the upper bounds of the misclassification costs [9].

A threshold adjustment is to modify the threshold or decision value, and there are some methods based on the rule of thumb [10], Fisher’s discriminant analysis [11], the midpoint between two-class data points using ensemble learning [12], and the $F_{1}$ score of the k-fold cross validation [13]. In order to evaluate the efficiency of classification, we consider the most time-consuming method proposed by Brank et al. (2003) [13], but we adjust the threshold based on the G-mean of the five-fold cross validation and rename it the CV-THR SVM.

Tuning the parameters is one of the most critical steps for training the model, and a grid search is the simplest method. However, it is time consuming to optimize the parameters of a nonlinear SVM by using a grid search [14,15]. Recently, many types of optimization algorithms were proposed to minimize the completion time, such as particle swarm optimization (PSO) [16,17,18], the genetic algorithm (GA) [15,16,18], a linear search [14], and others [19,20,21]. These optimization algorithms are all based on k-fold cross validation, and different metrics are used as the evaluation criteria. Taking k-fold cross validation as the fitness function may avoid overfitting but costs too much time, which is quite inefficient.

In this paper, we consider the SVM with a Gaussian radial basis kernel and C=10. In order to deal with the above problems, the imbalanced datasets and parameter selection, we purpose a fast and simple method based on a threshold adjustment, called b-SVM, to improve the classification performance for imbalanced datasets, and furthermore, we also propose an approach, called the Min-max gamma selection, to optimize the parameter γ of SVMs without carrying out an extensive k-fold cross validation. The remaining part of this paper is organized as follows: Section 2 describes our new methods, materials, and flowcharts. Section 3 presents the results of the experiments and compares our approaches with other methods. Section 4 is the discussions and conclusions.

## 2. Materials and Methods

### 2.1. b-SVM

To deal with the high rate of false negatives, we focus on the reasons that standard SVM $\hat{f}\left(x_{i}\right)$ formula easily becomes negative in the imbalanced dataset. First of all, we decompose and analyze the $\hat{f}\left(x_{i}\right)$ structure:

$$\begin{array}{cc} \; & \hat{f}\left(x_{i}\right)=\sum_{j=1}^{N}\alpha_{j}y_{j}K\left(x_{i},x_{j}\right)+b;\\ \; & b=\frac{1}{\#USV}\left(\sum_{i=1}^{\#USV}y_{i}-\phi {\left(x_{i}\right)}^{T}\beta \right);\\ \; & =\frac{1}{\#USV}(\left(\#PUSV\right)-\left(\#NUSV\right)-\\ & \sum_{i=1}^{\#USV}\sum_{j=1}^{N}\alpha_{j}y_{j}K\left(x_{i},x_{j}\right));\\ \; & =\Delta -\frac{1}{\#USV}\left(\sum_{i=1}^{\#USV}\sum_{j=1}^{N}\alpha_{j}y_{j}K\left(x_{i},x_{j}\right)\right), \end{array}$$

where USV are the support vectors whose α values are less than *C* and greater than 0, PUSV are the USV of minority class, NUSV are the USV of majority class, and tuning factor Δ:

$$\begin{array}{cc} \; & \Delta =\frac{\left(\#PUSV)-(\#NUSV\right)}{\#USV} \end{array}$$

Δ is directly related to the IR. In general, the number of support vectors in the majority class is larger than in the minority class, which implies that the more imbalanced the dataset is, the more negative Δ is. Because Δ results in the skewness of hyperplane, we correct the hyperplane by eliminating Δ, $\hat{f}\left(x_{i}\right)=\sum_{j=1}^{N}\alpha_{j}y_{j}K\left(x_{i},x_{j}\right)+b-\Delta$. Finally, b-SVM is defined as follows:

b-SVM:

$$\begin{array}{c} \hat{f}\left(x_{i}\right)=\sum_{j=1}^{N}\alpha_{j}y_{j}K\left(x_{i},x_{j}\right)+b; \end{array}$$

$$y_{i}=\left\{\begin{array}{cc} +1 & \;\mathrm{if}\;\hat{f}\left(x_{i}\right)>\Delta ;\\ -1 & \;\mathrm{if}\;\hat{f}\left(x_{i}\right)<\Delta , \end{array}\right.$$

### 2.2. Min-Max Gamma Selection

The two parameters, *C* and γ, play an important role in radial basis function kernel (Gaussian kernel) and there are no exact ranges for their values. The radial basis function kernel is defined as

$$K\left(x_{i},x_{j}\right)=\exp^{-\gamma {∥x_{i}-x_{j}∥}^{2}},$$

where γ is a parameter that sets how far the searching radius of training dataset reaches. Many researchers prefer applying k-fold cross validation to calculation of G-mean instead of training dataset as fitness values, to avoid overfitting or underfitting, even though the excessive completion time is required. In contrast, if we take the G-mean of training dataset as fitness value, it is usually to obtain the γ which is larger than the optimal γ. Although we can prevent the model underfitting and lower the completion time, overly large value of γ can easily cause model overfitting.

To overcome the problem above, a new method called Min-max gamma selection is proposed to select the appropriate γ value without carrying out k-fold cross validation. We select the γ from the set $\left\{2^{-20},2^{-19.5},\ldots ,\frac{1}{Data\;dimension}\right\}$ and calculate the “G-mean of training dataset” for each γ. In particular, we choose the smallest value of γ which has the largest G-mean of training dataset as the optimal γ, to avoid overfitting and underfitting. Min-max gamma selection Algorithm 1 is formally presented as follows:
**Algorithm 1:** Min-max gamma selection
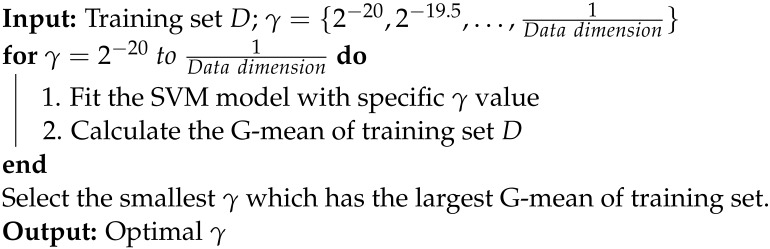


It is worth mentioning, *C*-value has effect on optimal γ-value, and based on our previous experiments, we suggest setting C=Datadimension or $C=\sqrt{Data\;dimension}$ for Min-max gamma selection.

### 2.3. Performance Measures

In order to evaluate classifiers on imbalanced datasets, using accuracy as a measure can be misleading. Therefore, we consider an alternative measure, G-mean, which is a measure of the ability of a classifier to balance sensitivity and specificity, has been widely used in imbalanced datasets, where G-mean $=\sqrt{sensitivity\times specificity}$, sensitivity $=\frac{TP}{TP+FN}$, and specificity $=\frac{TN}{TN+FP}$ (*TP* = true positive; *FN* = false negative; *TN* = true negative; *FP* = false positive). To evaluate whether the performance between two methods is significantly different, the paired-t test with significance level of α=0.05 is conducted for comparing paired classification results.

### 2.4. Simulation Study

We generate low-dimensional and high-dimensional datasets to evaluate the classification performance of SVMs. Low/(high)-dimensional datasets are generated as follows: Each observation has 30 (1000) features. Among all the features, 20 (900) are non-informative features, each following independently N(0,1) for both classes. The remaining 10 (100) are informative features following *k*-dimensional multivariate normal distributions $N_{k}\left(-\mu ,\Sigma \right)$ and $N_{k}\left(\mu ,\Sigma \right)$ for the majority class and the minority class, respectively, where *k* is the number of informative features, μ={0.25,0.5}, $\Sigma =\left(1-\rho \right)I_{k}+\rho 1_{k}1_{k}^{T}$, and ρ={0,0.7}. In this simulation, we assume that informative features are equi-correlated with correlation ρ and the degree of imbalance is quantified using the imbalance ratio (IR), which is represented as the ratio between the number of samples in the majority and minority classes. For each simulation experiment, we generate the training dataset of 60 and 200 samples, respectively, and the testing dataset of 2000 samples, with different IR={1,1.5,3}. In the end, we have 48 datasets, and each simulation is repeated 50 times. We use testing datasets to evaluate the classification performance of SVMs and ensure good statistical behavior.

### 2.5. Real Datasets

Six benchmark datasets are used to assess the performance of SVMs, and among all datasets, first two are low-dimensional datasets, and the rest of the datasets are high dimensional. Table 1 shows the summary of these real datasets. We use ten-fold cross validation and repeat this process 30 times to evaluate the classification performance.

### 2.6. Flow Chart for Experiments

**Figure 1:**
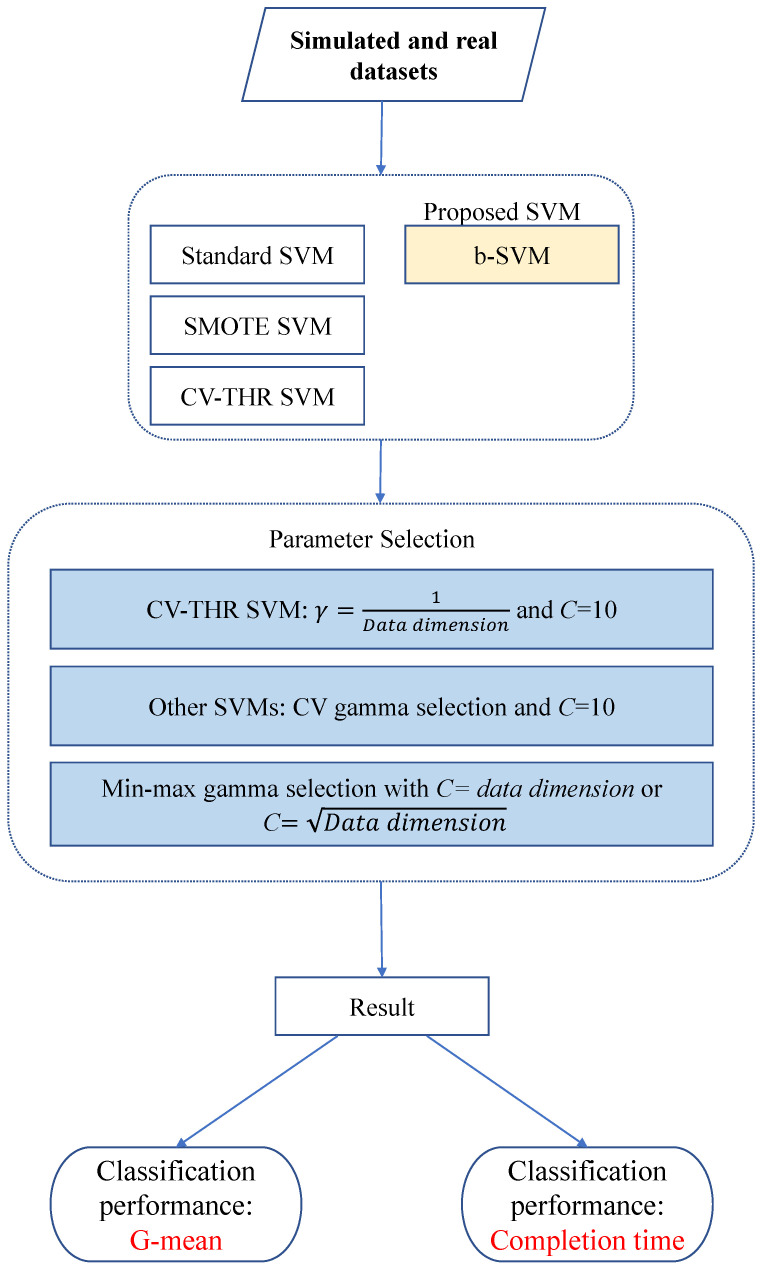
The flow chart of experimental design and analysis: parameter selection for imbalanced datasets.

First of all, we will compare our proposed method b-SVM with three conventional SVMs, standard SVM, SMOTE SVM, and CV-THR SVM, in a simulation study and real datasets. About parameter selection, we employed default C=10 and $\gamma =\frac{1}{Data\;dimension}$ for CV-THR SVM without carrying out parameter selection, because CV-THR SVM is too time-consuming. As for the remaining SVMs, we employed default C=10 and optimal γ based on five-fold cross validation (CV gamma selection). CV gamma selection is the most common and popular method to determine the parameter, even though it requires significant time to calculate. Second, with respect to gamma selection, we only consider three SVMs, standard SVM, SMOTE SVM, and b-SVM, and compare our approach Min-max gamma selection Algorithm 1 with the common method CV gamma selection. The flow chart is shown in Figure 1.

## 3. Results

### 3.1. Simulation Study

**Figure 2:**
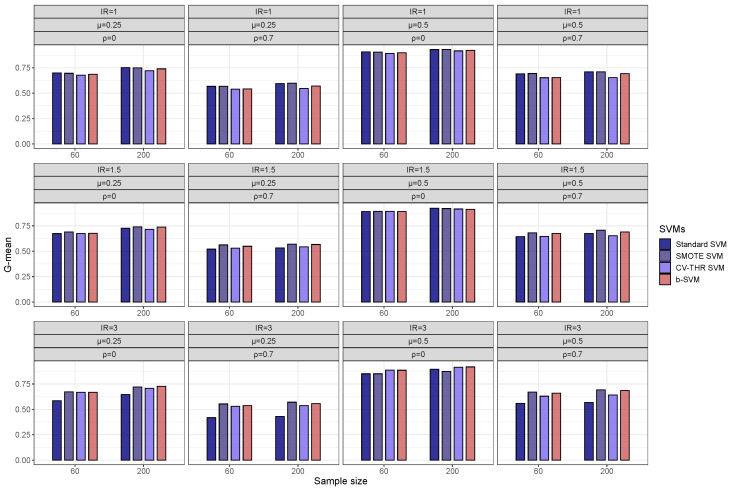
Classification performance (G-mean) of SVMs for simulated low-dimensional datasets.

**Figure 3:**
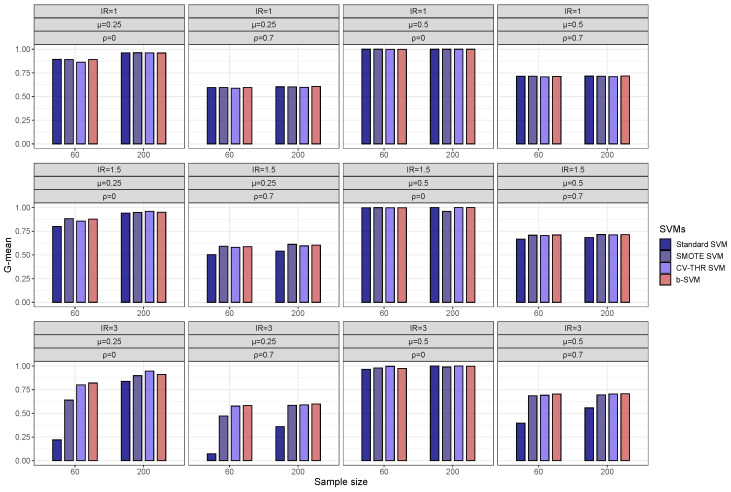
Classification performance (G-mean) of SVMs for simulated high-dimensional datasets.

Figure 2 shows the plots of the average G-means for the low-dimensional simulated data. When the class sizes are balanced, all four SVM methods achieve similar results and no significant difference exists between the four methods using pairwise comparisons. When the informative features are pairwisely correlated, the classification performance decreases with an inter-feature correlation and the standard SVM and SMOTE SVM appear to be slightly better than the other two methods. When the imbalance ratio (IR) increases, all three adjusting SVM methods can significantly improve the classification performance, particularly for correlated cases. Overall, the SMOTE SVM and b-SVM yield a better performance than the other methods and there is no significant difference between the SMOTE SVM and b-SVM. For the high-dimensional simulated data, Figure 3 shows that the general patterns of the classification performance are similar to those shown in Figure 2. However, the standard SVM performs much worse than the other three adjusting SVM methods when the class sizes are imbalanced. Another observation from Figure 3 reveals that the inter-feature correlation has a negative effect on the classification performance. Overall, the three adjusting methods yield a similar performance without a significant difference for all the scenarios, except in the cases where there is a little difference between two classes (μ=0.25), a higher imbalance ratio (IR = 3), and a smaller sample size (n=60). In such cases, the CV-THR SVM and b-SVM perform significantly better than the SMOTE SVM. In addition to improving the classification performance for the class imbalance data, the b-SVM has a much lower completion time, and in contrast, the SMOTE SVM and CV-THR SVM take over 1000 s to complete the procedures as shown in Table 2. Furthermore, it is notable that the CV-THR SVM has yet to carry out the parameter selection. In view of the classification performance and completion time, our proposed b-SVM adjusting method is the most efficient method and provides good classification performances across all 48 datasets.

**Figure 4:**
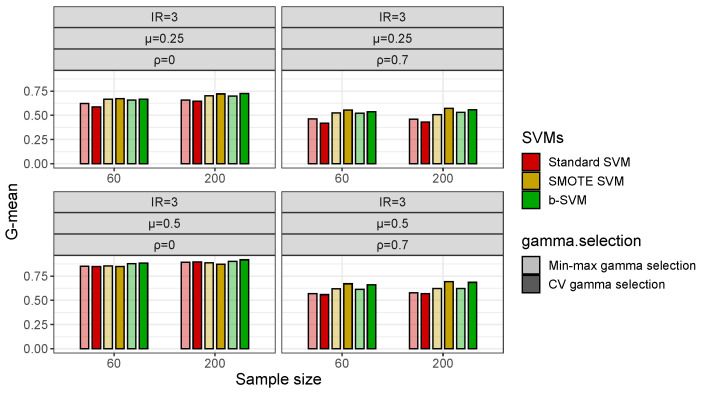
Classification performance (G-mean) of SVMs using different gamma selections in simulated low-dimensional datasets with IR=3.

**Figure 5:**
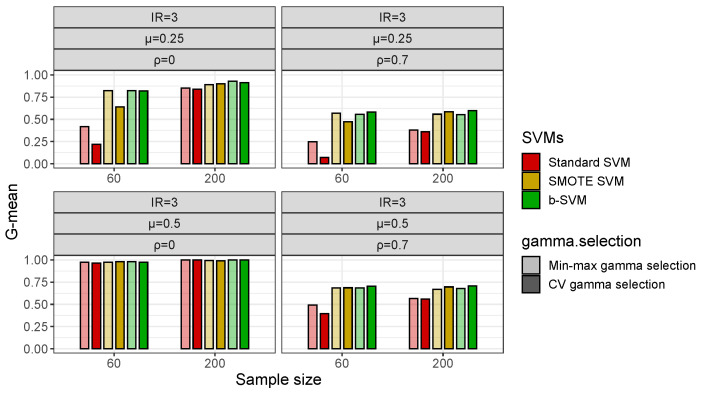
Classification performance (G-mean) of SVMs using different gamma selections in simulated high-dimensional datasets with IR=3.

With respect to the gamma selection, Figure 4 shows that in low-dimensional datasets with a class imbalance (IR = 3), the Min-max Algorithm 1 and CV gamma selection have a fairly close G-mean and there is no significant difference between the two gamma selection methods, but in some cases, the b-SVM using the Min-max gamma selection Algorithm 1 has a slightly lower value of the G-mean and these values are less than 2% different from their respective maximums. However, in high-dimensional datasets, the Min-max gamma selection Algorithm 1 significantly improves the performance of the standard SVM and SMOTE SVM when the inter-class effect is small (μ=0.25) and the G-mean is improved by more than 10% (Figure 5). Another observation from Figure 5 is that both gamma selection methods do not have a significant impact on the classification performance of the b-SVM. Furthermore, SVMs using the Min-max gamma selection Algorithm 1 require much less computation time, regardless of the low- and high-dimensional datasets (Table 3 and Table 4). Compared to the commonly used CV gamma selection, the Min-max gamma selection Algorithm 1 can provide a 70% to 80% reduction in the running time with no loss of the classification performance.

### 3.2. Real Datasets

In Table 5, the classification performance of the four SVM methods is compared in terms of the accuracy, G-mean, computation time (in seconds), and their standard deviation for the six real datasets. As expected, the standard SVM fails to predict the minority class, which results in a high accuracy and a low G-mean. In the low-dimensional datasets (Haberman and Liver), both the b-SVM and CV-THR SVM perform significantly better than the SMOTE-SVM and SVM in the G-mean. The G-mean is improved by almost 6–20%, while the CV-THR SVM is computationally less efficient and the b-SVM is more than 300 times faster than the CV-THR SVM. In the high-dimensional datasets, the b-SVM and CV-THR SVM can also provide a significant improvement in the G-mean. Moreover, in the Gastric tumor dataset, the b-SVM and CV-THR SVM provide significant improvements in the overall accuracy and G-mean simultaneously. However, the SMOTE-SVM does not present an improvement over the standard SVM for dealing with the class imbalance problem in the Glioma2 and Gastric tumor datasets. Here, again, the b-SVM can still reduce the computation time by up to 99% with no loss of the G-mean and accuracy.

**Figure 6:**
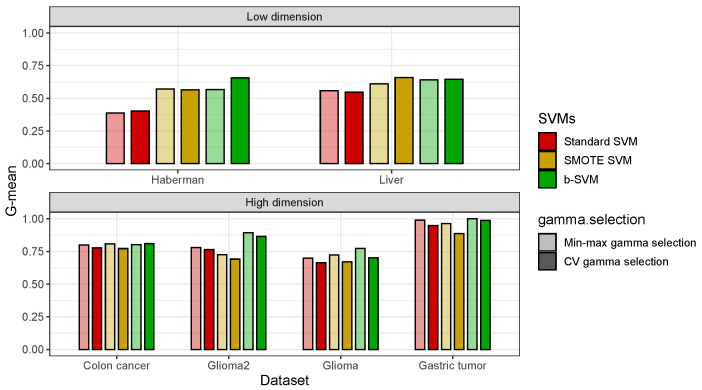
Classification performance (G-mean) of SVMs using different gamma selections in real datasets.

Next, we examine the performance of the different gamma selection methods combined with three SVM algorithms on the six real datasets. In Figure 6, the results reveal that the standard SVM and SMOTE SVM benefit the most from the combination of the Min-max gamma selection Algorithm 1 in the high-dimensional datasets. However, the b-SVM in combination with the Min-max gamma selection Algorithm 1 does not present an improvement in the low-dimensional datasets. In summary, although using the b-SVM in combination with the Min-max gamma Algorithm 1 selection may, in some cases, not provide a better performance than the CV gamma selection, the Min-max gamma selection Algorithm 1 can reduce the computation time by up to 65% with fairly close overall average classification performances as with the SMOTE SVM.

## 4. Conclusions

In this work, we have presented a novel threshold-adjusting method based on an SVM with a Gaussian radial kernel to deal with the class imbalance problem. The imbalance ratio is a critical factor that decreases the classification performance of the conventional SVM algorithms. Our simulation studies show that the classification performance of four SVM algorithms decrease statistically when the features are highly correlated with each other. In addition to the classification algorithm, the performance of most classifiers depends on the number of features and selected features. In such situations, a dataset may have redundant (features with a shared common predictive ability) and irrelevant (features providing no useful information) features. This implies that feature selection will help improve the classification performance by selecting the optimal set of features, especially in the datasets with many features (variables). In addition, the sample size shows a mild impact on the classification performance of both the b-SVM and CV-THR SVM as compared to the other SVM algorithms, while the b-SVM is computationally much less expensive than the CV-THR SVM. On the other hand, the classification performance of the conventional SVM is improved statistically by increasing the sample size.

Although the SVM performs well using default values in most cases, parameter optimization has a great impact on the classification performance of the SVM. Therefore, we also presented a novel gamma selection algorithm to find the optimal gamma parameter. The simulation and real data results show that all the adjusting SVM algorithms have a significant improvement for an imbalanced classification and our proposed b-SVM outperforms the other two SVM methods, both in terms of the G-mean and a reduction in the computation time. Another observation in this work is that the proposed Min-max gamma selection Algorithm 1 has been proven to be effective for SVM algorithms. When applied to six real datasets, the Min-max gamma selection Algorithm 1 can reduce the computation time by up to 65% with fairly close overall average classification performances as the respective maximum. In summary, the proposed b-SVM makes it possible to reduce the run time without a loss of the classification performance for handling an imbalanced classification. We also found that SVM algorithms may benefit from the Min-max gamma selection Algorithm 1 even though we observed less improvement in the low-dimensional real datasets. Our comparison study shows several interesting facts and provides the researchers some insights into the machine learning classifiers implementation on class imbalanced data. In the future, we plan to implement an extension of these workflows for multi-class classification problems.

## Figures and Tables

**Table 1:** Summary of real datasets used in the experiments.

Dataset	Features	#(+1)/#(−1)	Source
Haberman	3	81/225	[22]
Liver	5	105/240	[22]
Colon cancer	2000	22/40	[10,23]
Glioma2	4434	7/43	[24,25]
Glioma	4434	14/36	[24,25]
Gastric tumor	4522	8/22	[26]

**Table 2:** Completion time (s) of SVMs for simulated datasets.

SVMs	L.Datasets ^1^	H.Datasets ^2^
Standard SVM	474.14	887.09
SMOTE SVM	976.12	3277.14
CV-THR SVM	452.41	1011.20
b-SVM	474.85	902.50

^1^ L.Datasets: Low-dimensional datasets. ^2^ H.Datasets: High-dimensional datasets.

**Table 3:** Completion time (s) of SVMs using different gamma selections in simulated low datasets.

SVMs	Min-Max	CV
Standard SVM	127.47	474.14
SMOTE SVM	227.30	976.12
b-SVM	127.43	474.85

**Table 4:** Completion time (s) of SVMs using different gamma selections in simulated high datasets.

SVMs	Min-Max	CV
Standard SVM	258.92	887.09
SMOTE SVM	470.05	3277.14
b-SVM	257.05	902.50

**Table 5:** Mean (standard deviation in parentheses) classification performance of SVMs in real datasets.

		SVM			
Dataset	Metrics	Standard	SMOTE	CV-THR	b
Haberman	Accuracy	0.7213	0.6896	0.6423	0.7182
		(0.0020)	(0.0026)	(0.0045)	(0.0020)
	G-mean	0.3590	0.5393	0.5754	0.5974
		(0.0078)	(0.0056)	(0.0046)	(0.0056)
	Time (s)	17.18	22.84	5130.64	16.88
Liver	Accuracy	0.7489	0.6880	0.6879	0.7248
		(0.0015)	(0.0022)	(0.0030)	(0.0020)
	G-mean	0.5655	0.6152	0.6381	0.6298
		(0.0043)	(0.0034)	(0.0029)	(0.0031)
	Time (s)	19.55	31.58	6641.39	20.66
Colon cancer	Accuracy	0.8324	0.8403	0.8278	0.8307
		(0.0042)	(0.0037)	(0.0045)	(0.0038)
	G-mean	0.7797	0.7892	0.7949	0.8098
		(0.0092)	(0.0095)	(0.0115)	(0.0094)
	Time (s)	7.28	35.81	2997.96	6.73
Glioma2	Accuracy	0.9340	0.8133	0.8480	0.8540
		(0.0017)	(0.0096)	(0.0054)	(0.0045)
	G-mean	0.7874	0.6933	0.8236	0.8650
		(0.0093)	(0.0106)	(0.0116)	(0.0080)
	Time (s)	8.15	71.50	3330.43	7.89
Glioma	Accuracy	0.8587	0.8593	0.7940	0.8587
		(0.0027)	(0.0030)	(0.0084)	(0.0048)
	G-mean	0.6773	0.6797	0.6963	0.6825
		(0.0163)	(0.0165)	(0.0148)	(0.0163)
	Time (s)	7.90	71.56	3335.27	7.82
Gastric tumor	Accuracy	0.9011	0.8889	0.9456	0.9600
		(0.0049)	(0.0068)	(0.0040)	(0.0030)
	G-mean	0.8310	0.8132	0.9474	0.9647
		(0.0114)	(0.0125)	(0.0058)	(0.0053)
	Time (s)	7.60	66.89	3537.62	7.53

## Data Availability

Publicly available datasets were analyzed during this study. The data can be found in the Machine Learning Repository (http://archive.ics.uci.edu/ml, accessed on 12 December 2022). The R-codes used in this study are available from the corresponding author upon reasonable request.
